# Outlines of Medical Geography: Being an Inquiry How Far Calcareous Soils, or Strata, Counteract the Septic Exhalations, Which Occasion Distempers of a Febrile or Pestilential Type

**Published:** 1799-05

**Authors:** 


### *Outlines of Medical Geography: being an Inquiry how far Calcareous Soils, or Strata, counteract the Septic Exhalations, which occaſion Diſtempers of a febrile or peſtilential Type.*

[Extracted from a Letter of Dr. MITCHILL, of New York, to Dr. HAWORTH, of Oxford.]

IF there is any ſolidity in the obſervation made in my Letter to Dr. Beddoes of September 15, 1797, concerning the comparative mildneſs or rarity of peſtilential and febrile diſtempers in countries underlaid with extenſive ſtrata of ſuperficial *lime-ſtone*, as happens in ſome parts of the United States, then England ought to exhibit ſomething of the ſame kind in the counties where *chalk* is very prevalent. I now ſet myſelf down to recollect what I remarked about calcareous earth, in travelling from Dover to London, and during a walk I once took from London to Oxford and its environs, Woodſtock, Blenheim, and back again.

The chalk cliffs in the neighbourhood of Dover, are talked of by all perſons who navigate the channel, or who barely paſs from any of the oppoſite ports of France to this part of England. It has not been ſo generally noticed that the land from Folkſtone, in Kent, or thereabout, along by South Foreland, and almoſt to Deal, together with a good part ofthe Iſle of Thanet, including both Ramſgate and Margate, has a chalky bottom. Indeed, with very little interruption, the calcareous material prevails to a very conſiderable extent, on both ſides of the road from Dover to Canterbury, and thence to Rocheſter. In ſhort, I may ſay, it extends weſtward quite to the county of Surry, and paſſes into it by a tract almoſt as broad as the diſtance from Deptford to Weſterham. This is the moſt healthy part of the country, as may appear by comparing it with the unwholeſomeneſs of Oxney Iſle, Romney Marſh, and the Iſle of Sheppey, where not chalk, but ſiliceous ſand, flint, clay and loam, conſtitute the principal part of the ſoil. (See the Map prefixed to Mr. BOYS's General View of the Agriculture of the County of Kent. London, 1796.)

The chalk continues through part of Surry, Berkſhire, much of Oxfordſhire, quite into Glouceſterſhire, and prevails extenſively in Buckinghamſhire, Middleſex, and ſomewhat in Eſſex. Oxfordſhire, though ſituated ſo far inland, is famous, in many places, for its petrifactions and incruſtations of calcareous earth. Through this cretaceous land run the Iſis, the Charwall, the Windruſh, the Evenlade, the Thames, and, according to Dr. PLOT's enumeration, ſeventy other ſtreams of i[illegible]rior rank, whoſe collected waters paſs through through the enumerated chalky tracts to London, and the greater part of the diſtance thence to the ſea.

The inferences to be drawn from theſe geographical facts are two: 1ſt. That an extenſive body of chalk, or calcareous earth, of very conſiderable breadth, tinctures, with its peculiar qualities, the ſoil of England, from the German Ocean weſtward, to the hills, which ſeparate the waters of the Severn from thoſe of the Thames, in Glouceſterſhire; 2dly. That the greater part of all the ſtreams falling into the Thames, run through a country charged with calcareous earth, and conſequently muſt be conſiderably impregnated with that material.

With regard to the firſt point; if the *ſeptic gas* or *effluvium* is, as experiments lead us to believe, of an acid nature, in that caſe it ought not to be very abundant, or long prevalent, as a cauſe of epidemic, malignant, and peſtilential diſtempers, where the land is compoſed, in a good proportion, of calcareous earth; or, if in any place, or at any time, it ſhould happen that febrile diſorders, of greater or leſs malignity or inveteracy, break out, this ought to happen in conſequence of too much acid preſent for the chalk or lime to neutralize. On looking over the firſt authority that comes in my way, '*Martin's Natural Hiſtory of England*" (the edition publiſhed in 1759), I find Surry generally denominated "a pleaſant country;" Middleſex commended for its "exceedingly healthful air;" Berkſhire called "one of the moſt pleaſant counties in England;" Buckinghamſhire ſaid to poſſeſs an air generally good, eſpecially on the Chiltern hills: and, though the vale is dirty, "not ſo unhealthy as many other low lands in England, *the ſoil being marle or chalk*;" and the eaſtern part of Glouceſterſhire, where the Evenlaid and Windruſh rivers ariſe, though leſs fertile than the weſtern part of the county along the Severn, and more expoſed to wind and cold, "makes amends by its healthfulneſs."

The healthfulneſs of Oxfordſhire is almoſt proverbial; and the judicious choice of King ALFRED has long been commended for pitching upon ſo wholeſome a ſpot as that where the beautiful city of Oxford ſtands, as a ſeat for the Muſes. This has been conſidered as very ſtrikingly manifeſted by the two following circumſtances:—1ſt. An obſervation of long ſtanding, that although the ſmall-pox is as frequent there as any where elſe, the effects of it are ſeldom fatal; and 2d, that when the peſtilence, in 1665, was ſpread in a manner over the whole kingdom, though the court, both houſes of parliament, and the terms were held in the city of Oxford, yet the plague, notwithſtanding, was never there at all.—(1 Martin, 368.)

On the other hand, where, along the fenny hundreds of Eſſex, the joint operation of the current of the Thames, and the tides of the ocean, has brought brought together great bodies of fand and clay, that had been diffufed through the water, and been depofited by it, together with all fuch remains of animal and vegetable matter as conftitute the deep mould of thofe tracts, the country abounds thereabout with feptic exhalations, which are very injurious to the health of the inhabitants, where the neutralizing power of calcareous earth or chalk is wanting: and, as far as I can judge, a deficiency of the fame material enters deeply into the explanation of the unhealthinefs of the fens of Lincolnfhire, and the adjoining hundreds of Fleg and Merfh-land in Norfolk.

It muft be obferved, however, although chalky and calcareous foils are thus deftructive of that kind of air or vapour which produces agues, fevers, &c. that it does not follow, by the rule of contraries, that all fandy, loamy, gravelly, and clayey foils, muft be unhealthy. This may, however, be remarked, that in the United States of America, the moft fickly parts are the tracts along the Atlantic, where the land confifts in pretty much the whole range from New York to Florida, of filicious fand and clay, varioufly mingled, and tempered with more or lefs of mould, without any confiderable admixture of calcareous earth; and this, when it occurs, confifting princi-cipally of concretions of marine fhells, dug up in fome parts of Virginia, and the other fouthern ftates. As to our cities, the fite of New York is a fandy loam or gravel, except that part where the *plague* has ufually prevailed hitherto, which is built upon falt meadow, miry fwamp, and rotten trafh. Philadelphia ftands partly upon a fandy, and partly upon a ftiff loamy or brick-earth, impermeable to water, and deftitute of a fufficient portion of calcareous earth to keep down and attach peftilential fluids. Of both thefe cities it may be remarked with truth, that although they were built in a bottom of *lime*, or *chalk itfelf*, there are local caufes enough to engender the worft forms of diftempers, as happened at the famous affizes in Oxford, where the filth accumulated around the wretched criminals in prifon, generated peftilential matter enough to poifon a confiderable number of the court and attendants.

As far as I can comprehend the fubject, the general conclufion is this:—countries abounding with calcareous earth, are moftly free from the ravages of wide-fpreading epidemics, by reafon of the power that material poffeffes of neutralifing the acid of putrefaction; though, from particular local caufes, peftilence may be manufactured in places thus favourably circumftanced: while thofe places, which are moft remarkable for the prevalence of malig-nant and peftilential difeafes of that kind, confift of a foil through which chalk and lime are fcantily fcattered. England affords abundant proof of the former; the United States, of the latter obfervation.

To

To proceed now to the ſecond point. The numerous ſtreams of water, whoſe united body forms the Thames, run principally, it was ſaid, through a tract of country, plentifully furniſhed with chalk. The form in which this calcareous earth is moſt prevalent, is that of carbonat, or in combination with fixed air, and capable in many places of being burnt to quick-lime: though, doubtleſs, where the ſeptic (nitric) acid exiſts, it unites with the earthy baſis into calcareous nitre. There is, unqueſtionably, ſomewhat of a muriat of lime, eſpecially below London-bridge, and the whole diſtance thence, through the brackiſh and ſalt water, to the ocean. And perhaps there may be, in ſome places, a combination of the ſulphuric acid with the calcareous matter into gypſum. The ſmall quantity of gypſum which exiſts there, and if much did exiſt, the very trifling proportion of it which water is capable of diſſolving, leave very little to be ſaid concerning it here. The muriat of lime is there probably in too ſmall proportion to be matter of conſequence, any where above the flow of the ſalt water; and though, as Camden ſays, the river ſwells as high as Richmond, which is ſixty miles inland (*Britannia*, 187) with the tide, yet the ſalt reaches no great diſtance above Graveſend.

The ſeptite of lime is ſoluble in water: but, as it is alſo a nutritive ingredient in manures, and eaſy of reſolution into its conſtituent parts by plants, it is preſumable that a large proportion of what is formed of that compound, undergoes decompoſition on the ſoil where it is produced. The carbonat of lime, then, or common mild chalk, is the material upon which the ſtreams of water, in the caſe before us, muſt principally act.

Water may work upon this mild calcareous earth in two ways: 1. by acting as a menſtruum, or jointly with ſome ſubſtance which is a menſtruum, and *chemically* diſſolving it; and, 2, by *mechanical* attrition, wearing it away, reducing it to fine particles, and while it is diffuſed and floating through the liquid, carrying it down in its proper form, to be mingled and depoſited with the various matter of intervale ſpots, alluvial ſhores, and ſecondary iſlands.

It is hard to determine what quantity of calcareous earth is brought down in theſe two modes, from the adjacent counties into the bed of the Thames, and hurried along with his ſtream towards its diſemboguement. When all circumſtances of ſhowers, rains, freſhets, and the unceaſing atteration of the ſtreams along their channels, are taken into the account, the quantity would ſeem to be very conſiderable. This chalky ſubſtance, whether diſſolved in the water, or diffuſed through it, cannot fail to modify and influence it remarkably; in an eſpecial manner contributing to fertilize the low lands in the neighbourhood of the river, which it viſits or overflows.

Thus the ſeptic and other acids with which the Thames water was charged, are in a great meaſure, or perhaps quite, neutralized by the calcareous earth before it reaches London. The quality of the water there will be in a good degree determined, then, by the materials furniſhed by that city. The acid of putrefaction and other acids running into it from the ſewers, will, under the exiſting regulations, be neutralized by the refuſe alkaline matter of houſe-keeping, and other conſumptive proceſſes: and the pot-aſh and ſoda thrown away in ſoap, &c. ſeem to more than counterbalance their opponents, and give a preponderating influence to the alkalis. Hence evidently proceeds the boaſted ſoftneſs of the Thames water, for the waſhing of linen; for enabling the London dyers to ſtrike their bright and laſting colours; for keeping ſo well at ſea as it does; and probably enabling it to extract, in a very complete manner, the ſubſtance of malt, to form their excellent porter. Is not all this confirmed by the fact known to navigators, that the Thames water, after long keeping, depoſits a ſtony ſediment on the bottoms of the caſks?

That fertile tract of land, the Carſe of Gowrie, in the county of Perth, in Scotland, which is reckoned to poſſeſs a climate more mild and favourable to vegetation than any part of that kingdom, affords direct evidence of the healthineſs conſequent upon uſing lime as a manure. The ſoil conſiſts chiefly of rich clay, loam, and ſharp gravel; and the inhabitants, until the year 1735, uſed to be ſubject to the ague. Then, one or two of the principal proprietors undertook, by draining, ſummer-fallowing, and ſowing graſs ſeeds, to improve their eſtates. Accident led them to a diſcovery of the efficacy of lime on that ſoil, from obſerving the powerful effects of ſome old lime rubbiſh of decayed buildings, when ſpread on the corner of a field. The liming their lands then came gradually into uſe, and has ſince been generally adopted; the conſequence of which is, *the ague has long diſappeared*. Here ſeems to have been a beautiful experiment made upon about ninety-ſix ſquare miles of country, where the ſeptic ſteams that formerly gave the people agues, are now attracted by the lime, and turned to calcareous nitre; while increaſed productiveneſs of the land, and greater wholeſomeneſs of the air, continue to be the happy conſequences. *(Donaldſon's General View, &c. p. 12, et ſeq.)* Some judgment may hence be formed, concerning the power of art in changing the face of nature. What a grand reflection, that an inconſiderable quantity of powdered lime ſtrewed over the land, ſhould thus coerce the matter of peſtilence, and control the operations of the atmoſphere!

In Linnæus's hypotheſis *(I. Amœnitat. Academic.)* on the cauſe of intermittent fevers, we find a collection of facts to prove their connection with argillaceous earth, or clayey ſoil. Of this he was ſo well ſatisfied, that he he concluded, that attenuated particles of clay, taken into the body with food and drink, entered the blood, ſtuck in the extreme branches of the arteries, and brought on, as a true proximate cauſe, the ſymptoms of the diſeaſe.— (Hypotheſis nova, § v.) The ſenſible inquirer will find, in his fourth ſection, an enumeration of all the parts of Sweden famous for intermittents, and for ſtrata of argillaceous ſoil, and the authority of Mr. SANDEL, quoted as an eye-witneſs of the ſame coincidence of clayey bottoms and intermittent fevers in Penſylvania. The facts I take to be indubitable. Linnæus has reaſoned upon the ſubject, by conſidering argillaceous earth alone. I have viewed it in contraſt with calcareous earth, that, by embracing a wider range of facts, the operation of the latter, in tempering the former, may be the better comprehended. Whether my theory is better founded than that of the Profeſſor of Upſal, the experienced and candid will judge.

